# Age-Related Decrease in Abdominal Pain and Associated Structural- and Functional Mechanisms: An Exploratory Study in Healthy Individuals and Irritable Bowel Syndrome Patients

**DOI:** 10.3389/fphar.2021.806002

**Published:** 2021-12-16

**Authors:** Abraham B. Beckers, Ellen Wilms, Zlatan Mujagic, Béla Kajtár, Kata Csekő, Zsa Zsa R. M. Weerts, Lisa Vork, Freddy J. Troost, Joanna W. Kruimel, José M. Conchillo, Zsuzsanna Helyes, Ad A. M. Masclee, Daniel Keszthelyi, Daisy M. A. E. Jonkers

**Affiliations:** ^1^ Division of Gastroenterology and Hepatology, Department of Internal Medicine, NUTRIM School for Nutrition and Translational Research in Metabolism, Maastricht University Medical Center+, Maastricht, Netherlands; ^2^ Department of Pathology, Clinical Centre, Medical School, University of Pecs, Pécs, Hungary; ^3^ Department of Pharmacology and Pharmacotherapy, Medical School and Molecular Pharmacology Research Group, Szentágothai Research Centre, University of Pecs, Pécs, Hungary; ^4^ PharmInVivo Ltd, Pécs, Hungary

**Keywords:** ageing, visceral nociception, TRPV1, TRPA1, mRNA, immunofluorescence microscopy

## Abstract

**Introduction:** The world population is ageing, resulting in increased prevalence of age-related comorbidities and healthcare costs. Limited data are available on intestinal health in elderly populations. Structural and functional changes, including altered visceroperception, may lead to altered bowel habits and abdominal symptoms in healthy individuals and irritable bowel syndrome (IBS) patients. Our aim was to explore age-related changes in gastrointestinal symptoms and underlying mechanisms.

**Methods:** In total, 780 subjects (IBS patients *n* = 463, healthy subjects *n* = 317) from two separate studies were included. Subjects were divided into different age groups ranging from young adult to elderly. Demographics and gastrointestinal symptom scores were collected from all participants using validated questionnaires. A subset of 233 IBS patients and 103 controls underwent a rectal barostat procedure to assess visceral hypersensitivity. Sigmoid biopsies were obtained from 10 healthy young adults and 10 healthy elderly. Expression of the visceral pain-associated receptors transient receptor potential (TRP) Ankyrin 1 (TRPA1) and Vanilloid 1 (TRPV1) genes were investigated by quantitative RT-PCR and immunofluorescence.

**Results:** Both elderly IBS and healthy individuals showed significantly lower scores for abdominal pain (*p* < 0.001) and indigestion (*p* < 0.05) as compared to respective young adults. Visceral hypersensitivity was less common in elderly than young IBS patients (*p* < 0.001). Relative TRPA1 gene transcription, as well as TRPA1 and TRPV1 immunoreactivity were significantly lower in healthy elderly versus healthy young adults (*p* < 0.05).

**Conclusions:** Our findings show an age-related decrease in abdominal pain perception. This may in part be related to decreased TRPA1 and/or TRPV1 receptor expression. Further studies are needed to reveal precise underlying mechanisms and the associations with intestinal health.

## Introduction

Ageing affects gut functioning on several levels; for example motility, gut microbiota composition, local immune and inflammatory responses and sensory functions (e.g. gustatory and nociceptive) (Soenen et al., 2016; DeJong et al., 2020) are reported to change with age. Perturbations in these functions can be linked to common gastrointestinal (GI) disorders in the elderly: for example constipation and diverticulitis occur more frequently with ageing (Higgins and Johanson, 2004; Hays and Roberts, 2006). In addition, acute inflammatory gastrointestinal diseases, such as appendicitis and cholecystitis, can be more difficult to diagnose as their presentation in the elderly is more often atypical or silent, in part related to less severe abdominal pain (Lyon and Clark, 2006). On the other hand, decreased nociceptive signaling could be of benefit in disorders of brain-gut interactions that are characterized by chronic recurrent pain, such as irritable bowel syndrome (IBS). Studies using balloon distentions in the esophagus and rectum demonstrated an increased visceral pain threshold in the elderly (Lasch et al., 1997; Lagier et al., 1999), and abdominal pain was shown to be inversely correlated with age during a nutrient challenge in the form of enteral feeding solution (Gururatsakul et al., 2010). The decreased sensitivity to visceral pain appears to translate to lower prevalence rates of IBS, as a meta-analysis showed that the odds of IBS in those aged 50 years and older are significantly lower than in those younger than 50 years (Lovell and Ford, 2012). Moreover, two population-based studies demonstrated the disappearance of abdominal pain with ageing (Choung et al., 2014; Rottenberg et al., 2015).

Currently, little is known about the mechanisms behind the decline in sensory signaling in the gut with increasing age. Elucidating these mechanisms could potentially provide new insights for the development of novel (visceral) pain management strategies, in particular with respect to different therapeutic responses related to age. From a molecular perspective, the responsiveness of visceral afferents to noxious stimuli is determined by the expression and activity of sensory transducer molecules including transient receptor potential (TRP) channels (Beckers et al., 2017). TRP channels constitute a family of nonselective cation channels. Several members of this family, including transient receptor potential vanilloid 1 (TRPV1) and transient receptor potential ankyrin 1 (TRPA1), have been identified to function as transducers of nociceptive signals in both somatic and visceral pain (Beckers et al., 2017). It has therefore been hypothesized that a reduction in functioning or expression of TRPV1 and TRPA1 in visceral afferents is responsible for reduced abdominal pain with age. Significantly decreased responses to the TRPV1 agonist capsaicin in colonic high threshold mechanosensitive afferents was observed in 24-month-old mice when compared to 3-month-old ones, which were unrelated to the expression of TRPV1 in DRG neurons (Keating et al., 2016). Similarly, in human ileal and sigmoid biopsy samples, a decreased baseline firing rate and blunted response to bradykinin were shown in elderly compared to young individuals (with a cut-off of 65 years of age) (Yu et al., 2016).

Given the limited data with regards to alterations in TRP channel expression, signaling and function with ageing and abdominal pain, our aim was to explore age-related changes in gastrointestinal symptoms including nociception, and particularly their relationship with TRPV1 and TRPA1 immunoreactivity and mRNA expression profiles in sigmoid biopsies. In addition to subjective abdominal symptom ratings we studied visceral sensitivity across age groups using rectal balloon distension in IBS patients and healthy controls.

## Methods

The present manuscript is based on data from two separate studies. One study was part of a larger project on the effect of pectin on GI function, hereafter referred to as the ‘biopsy study’ (An et al., 2019; Wilms et al., 2019). This involved a randomized, double-blind, placebo-controlled trial in healthy young adults (18–40 years) and healthy elderly (65–75 years), which had been registered in the US National Library of Medicine (NCT02376270). The second study is part of a larger cohort study on the phenotypic and genotypic characterization of IBS patients, hereafter referred to as the ‘Maastricht IBS (MIBS) cohort’ (Ludidi et al., 2014; Mujagic et al., 2017), which is registered in the US National Library of Medicine (NCT00775060). Subjects aged between 18 and 75 years were included in this primay-tertiary care cohort study. IBS was diagnosed using the Rome III criteria in all patients. Fulfilment of criteria was checked by means of interview by an experienced clinical researcher (medical doctor or last year medical doctor in training). In addition, healthy controls (i.e. no current or past gastrointestinal disorders) were included in this cohort study. Patient characteristics of the Maastricht IBS cohort, including symptom scores, intestinal permeability, serotonin metabolism and visceral hypersensitivity has been previously reported elsewhere (Ludidi et al., 2014; Mujagic et al., 2016; Thijssen et al., 2016; Mujagic et al., 2017; Vork et al., 2018; Weerts et al., 2019). The current exploratory study was specifically aimed at investigating age-related changes. Both studies had been approved by the Maastricht University Medical Center+ (MUMC+) Ethics Committee. All study procedures were performed in compliance with Good Clinical Practice Guidelines and according to the revised Declaration of Helsinki. All subjects gave written informed consent prior to participation. An overview of the obtained data per study and sample sizes is provided in Table 1. Details on the methods are provided below.

**TABLE 1:** Overview of available data.

Healthy subjects (biopsy study)	IBS patients and healthy controls (maastricht IBS cohort)	
18–40 years: Young adults (*n* = 52)	*IBS patients*	*Healthy controls*
65–75 years: Elderly (*n* = 48)	18–39 years: Young adults (*n* = 191)	18–39 years: Young adults (*n* = 90)
	40–64 years: Middle-aged adults (*n* = 209)	40–64 years: Middle-aged adults (*n* = 84)
	65–75 years: Elderly (*n* = 63)	65–75 years: Elderly (*n* = 43)
Gastrointestinal Symptom Rating Scale (*n* = 100)	Gastrointestinal Symptom Rating Scale (*n* = 463 IBS patients, *n* = 217 healthy controls)	
Visceral pain-associated parameters (sigmoid biopsies)	Visceral hypersensitivity (rectal barostat procedure;*n* = 233 IBS patients, *n* = 103 healthy controls)	
- TRPA1 and TRPV1 gene mRNA expression		
(*n* = 20)				
- TRPA1 and TRPV1 nerve fibre stainings (*n* = 16)				

### Gastrointestinal Symptom Rating Scale Questionnaire

The GSRS was completed by all subjects in both studies. This 15-item questionnaire encompasses a range of gastrointestinal symptoms, including abdominal pain, bloating, flatulence, frequency of bowel movements, stool consistency, urgency, and the feeling of incomplete evacuation (Svedlund et al., 1988). Symptoms are combined into five clusters, which include abdominal pain, reflux, diarrhoea, indigestion and constipation. The intensity of symptoms is scored on a 7-grade Likert scale (1, no symptoms at all; 2, minimal symptoms; 3, mild symptoms; 4, moderate symptoms; 5, rather serious symptoms; 6, serious symptoms; and 7, very severe symptoms).

### Sigmoid Biopsies–TRPA1 and TRPV1 Analyses

In the biopsy study, tissue samples were obtained from the sigmoid colon by means of standard flexible sigmoidoscopy and snap frozen in liquid nitrogen upon storage at -80C until further analysis. No bowel preparation was used. Biopsies were obtained after the supplementation period, and only subjects receiving placebo were included for TRP analyses. Note that placebo consisted of the polysaccharide maltodextrin. Maltodextrin is completely digested and absorbed in the small intestine and therefore has no significant effect on the colon (Leemhuis et al., 2014).

### TRPV1 and TRPA1 mRNA Expression

Sample homogenisation of biopsy specimen was performed in 1 ml TRI Reagent (Molecular ResearchCentre Inc., Cincinatti, OH, United States) and total RNA was isolated with Direct-Zol RNA MiniPrep isolation kit (Zymo Research, Irvine, CA) following the manufacturer’s protocol. Samples were then measured by NanoDrop ND-1000 spectrophotometer (NanoDrop Technologies Inc., Wilmington, DE) to assess RNA quantity and purity. After treatment with deoxyribonuclease I enzyme (Zymo Research, Irvine,CA) total RNA (100 ng) was reverse transcribed with Maxima First Strand cDNA Synthesis Kit (Thermo Fisher Scientific, Waltham, MA) according to the manufacturer’s instructions. To amplify trancripts, real-time qPCR was performed on a Stratagene Mx3000P qPCRSystem (Agilent Technologies, Santa Clara, CA) using Luminaris HiGreen LowROX qPCR Master Mix (Thermo Fisher Scientific) (Bohonyi et al., 2017). The following primer pairs were used to amplify the genes of interest: Trpv1 (NM_080706.3) (sense): 5′-CAG​CTC​AAT​TGC​TGT​GCA​GGT​TA-3′ and (antisense): 5′-TGC​CAG​TAT​GGA​TGG​AGT​GGA​A-3'; Trpa1 (NM_007332.2) (sense): 5′-ATG​GAC​AGC​TTG​GTT​ACC​TCC​AC-3′ and (antisense): 5′- CAG​CAC​TCT​GCT​GGT​TTG​TAT​GAA -3'. All reactions were measured in triplicates and the geometric mean of their Ct values were calculated which was normalized to transcripts of the glyceraldehyde 3-phosphate dehydrogenase (Gapdh) (NM_001289746.1) (sense): 5ʹ- CCT​GCA​CCA​CCA​ACT​GCT​TA -3ʹ and (antisense): 5ʹ-TAG​AGG​CAG​GGA​TGA​TGT​TCT​G-3ʹ; used as reference gene. Primers with similar efficiencies were used and melt curve analyses were performed to verify primer specificity. The determination of relative messenger RNA (mRNA) expression levels was performed according to the comparative Ct method.

### TRPV1 and TRPA1 Immunofluorescent Staining

Five µm frozen sections of sigmoid biopsies were fixed on Superfrost Plus Microscope Slides (Thermo Fischer Scientific, Waltham, United States) and stained with a 1:100 dilution of guinea pig polyclonal anti-TRPV1 (GP14100, Neuromics, Edina, MN, United States) and 1:200 dilution of rabbit polyclonal anti-TRPA1 (orb86362, Biorbyt, Cambridge, United Kingdom) primary antibodies. Slides were incubated with VECTASTAIN^®^ ABC-Peroxidase Kit- Guinea Pig IgG (PK-4007, BioMarker Ltd., Budapest, Hungary) and HISTOLS peroxidase kit - rabbit IgG (30011.R500 Histopathology Ltd, Pécs, Hungary). The reaction was then visualized by 1:2000 dilution of Tyramide Signal amplification kit with HRP-goat anti-rabbit IgG and Alexa Fluor 594 and 488 tyramide (T20925 and T20922, respectively; Life Technologies, Paisley, UK). Nuclear counterstaining for fluorescent microscopy was performed by Vectashield Mounting Medium with 4′,6-diamidino-2-phenylindole (DAPI) in 1.5 ug/mL concentration (Vector Laboratories, Burlingame, CA, United States). TRPV1 and TRPA1 immunopositivity was quantified by counting TRPA1 and TRPV1 positive nerve fiber-like structures per area of lamina propria performed by an experienced pathologist who was blind to the treatment order (BK). Incubating sigmoid mucosa with Tris-buffered saline instead of the primary antibodies served as negative control, while sections of previously sampled human dorsal root ganglia expressing TRPV1 and TRPA1 abundantly were used as positive control. The antibody specificity has been validated by preabsorption of the respective blocking peptides (P14100 Neuromics, Edina, MN, United States for TRPV1 and AAP35205 Aviva Systems Biology, San Diego, CA, United States) as described previously (Kun et al., 2014).

### Rectal Barostat (Balloon Distension)

Participants arrived in the hospital after an overnight fast. Rectal perception was measured using an electronic barostat (Distender II; G&J Electronics, Toronto, ON, Canada, part: C7-CB-R) and a balloon of non-compliant material (Mui Scientific, Missisauga, ON, Canada, part: C7-2CB-R), which was lubricated with KY-gel (Johnsson and Joshnsson, Longhorne, PA, United States). The procedure was executed according to our standardised protocol as described previously (Ludidi et al., 2012). Abdominal pain was scored using a visual analogue scale (VAS) ranging from 0 to 100 mm. For subjects who did not complete the full protocol, VAS-scores for the remaining pressure steps were padded using the highest VAS-score achieved over the protocol thus far. Visceral hypersensitivity was defined as a VAS-score of ≥20 mm at a pressure of 26 mmHg or lower.

### Data Visualization and Statistical Analysis

All data plots were created using GraphPad Prism 9. Barcharts with error bars include superimposed individual data points. Tabular data are presented as mean ± standard deviation (SD) for continuous variables and proportions for categorical outcomes. Continuous variables were found to be normally distributed. Statistical analyses were performed in R Statistical Software version 3.6.3 (2020–02–29). Univariate comparisons were performed using an independent samples *t*-test for continuous variables and χ^2^ test for dichotomous variables. A two-sided alpha value of 0.05 was used. Given the hypothesis-generating/exploratory nature of the current study, significance level was not corrected for multiple comparisons.

## Results

### Subject Characteristics

The baseline characteristics of subjects from both studies are displayed in Table 2. In the healthy subject subgroup of the MIBS cohort, young and middle-aged subjects were more often female than elderly subjects. Gender was equally distributed in IBS patients (Maastricht IBS cohort) and healthy subjects from the biopsy study. A significantly higher proportion of healthy young adults and middle-aged subjects in the Maastricht IBS cohort was female, as compared to healthy elderly. The BMI of elderly subjects was significantly higher across all groups, as compared to their younger counterparts. Proton pump inhibitors were more frequently used by elderly subjects in the IBS patient subgroup, as well as in the biopsy study, as compared to the respective young subjects. Selective serotonin reuptake inhibitors were more frequently used by middle-aged IBS patients, as compared to elderly IBS patients.

**TABLE 2:** Baseline characteristics of IBS patients (MIBS cohort) and healthy subjects (MIBS cohort and biopsy study).

	IBS patients (MIBS)			Healthy subjects (MIBS)			Healthy subjects (biopsy study)	
	Young adults	Middle-aged	Elderly	Young adults	Middle-aged	Elderly	Young adults	Elderly
N	191	209	63	90	84	43	52	48
Age (yrs, mean ± SD)	26.9 ± 6.1	52.5 ± 7.2	70.3 ± 4.1	23.9 ± 4.1	55.1 ± 7.4	68.4 ± 2.5	23.1 ± 4.3	69.7 ± 2.8
Female (%)	77.0	72.2	65.1	72.2**	60.7*	41.9	57.7	43.7
BMI (kg/m^2^, mean ± SD)	23.7 ± 4.9***	25.7 ± 4.3	26.4 ± 4.1	22.5 ± 2.9**	25.2 ± 4.2	25.0 ± 3.4	22.9 ± 2.7***	25.8 ± 2.7
Symptom duration (yrs, mean ± SD)	7.2 ± 6.9***	17.3 ± 15.7	21.6 ± 18.7	N.A.	N.A.	N.A.	N.A.	N.A.
IBS subtype (%)								
IBS-C	17.2	21.3	21.3	N.A.	N.A.	N.A.	N.A.	N.A.
IBS-D	33.9	36.6	27.9					
IBS-M	45.2	34.7	45.9					
IBS-U	3.8	7.4	4.9					
Medication (%)								
PPI	11.5***	33.0	30.2	1.1	3.6	7.0	0**	12.5
NSAID	24.1*	28.7	38.1	13.3	21.4	25.6	-	-
SSRI	9.4	17.2*	6.3	2.2	2.4	4.7	-	-
Motility + drugs	17.8	18.2	23.8	0	0	0	-	-
Motility - drugs	14.1	16.3	12.7	0	0	0	-	-

* *p* < 0.05 ** *p* < 0.01 *** *p* < 0.001 (*vs*. elderly for MIBS), SD = standard deviation.

Independent t-tests were used for continuous variables, chi-sqaured tests for proportions.

Abbreviations: IBS-C, IBS constipation predominant subtype; IBS-D, IBS diarrhoea predominant subtype; IBS-M, IBS mixed subtype; IBS-U, IBS unspecified subtype; PPI, proton pump inhibitor; NSAID, non-steroidal anti-inflammatory drug; SSRI, selective serotonin reuptake inhibitor.

Motility + drugs: e.g., polyethylene glycol, motility—drugs: e.g., loperamide.

MIBS age groups: 18–39 years (young adults), 40–64 years (middle-aged adults), 65–75 years (elderly).

Biopsy study age groups: 40–64 years (middle-aged adults), 65–75 years (elderly).

### Abdominal Symptoms and Visceral Hypersensitivity

**FIGURE 1:**
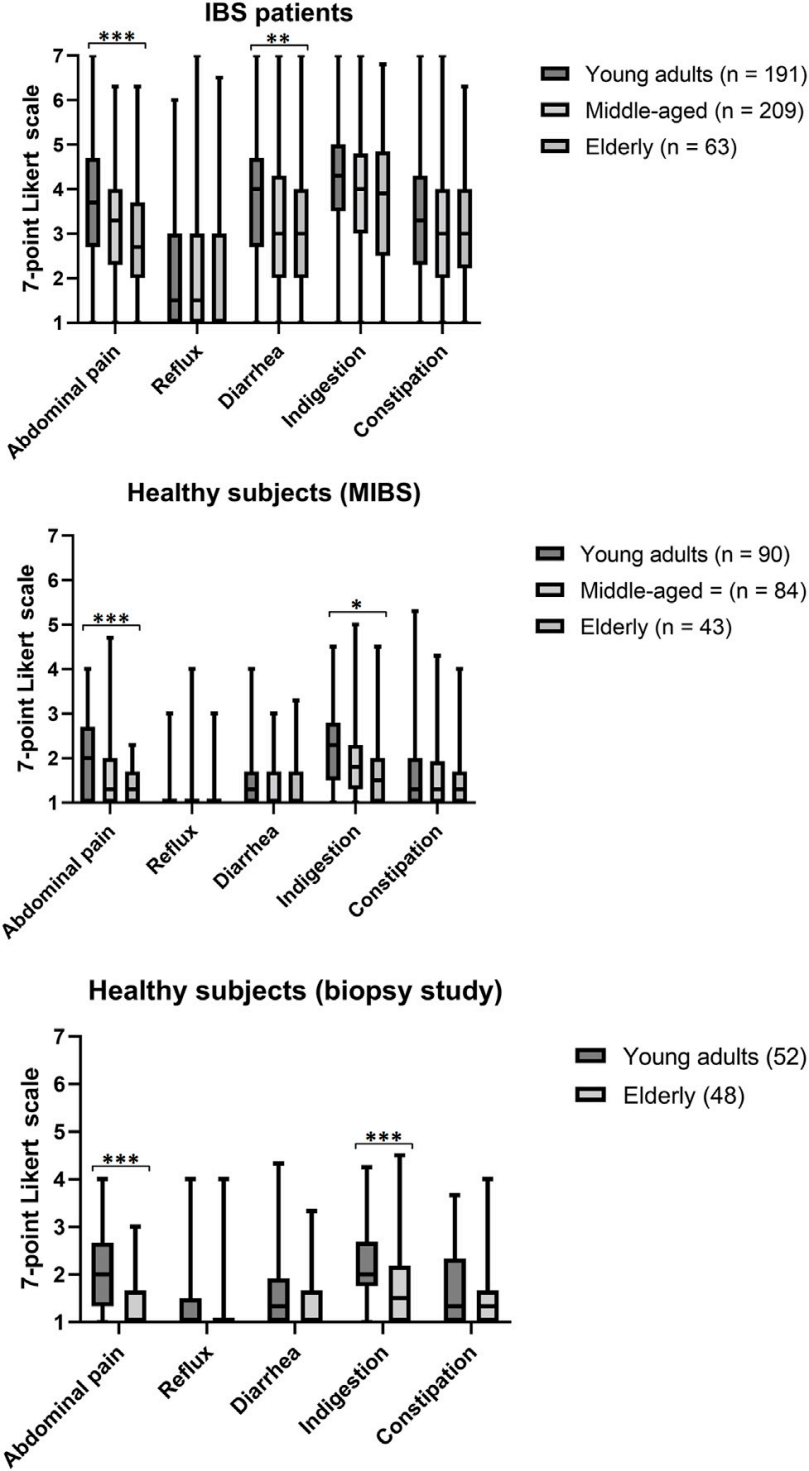
Boxplot showing GSRS scores in IBS patients panel **(A** **)**, healthy subjects from the Maastricht IBS cohort study panel **(B)** and healthy subjects from the biopsy study panel **(C).** Plots include median, interquartile range and minimum and maximum values. Independent t-tests were used for comparison of mean GSRS scores per age group. *p < 0.05 ** *p* < 0.01 *** *p* < 0.001.

Median and mean GSRS scores are shown in Figure 1; Supplementary Table S1, respectively. Scores for abdominal pain were significantly lower in elderly as compared to young subjects in all groups (in both IBS patients and healthy subjects). In IBS patients, scores for diarrhoea were also lower in elderly as compared to young subjects. In healthy subjects from both the Maastricht IBS cohort and the biopsy study, scores for indigestion were lower among elderly subjects.

**FIGURE 2:**
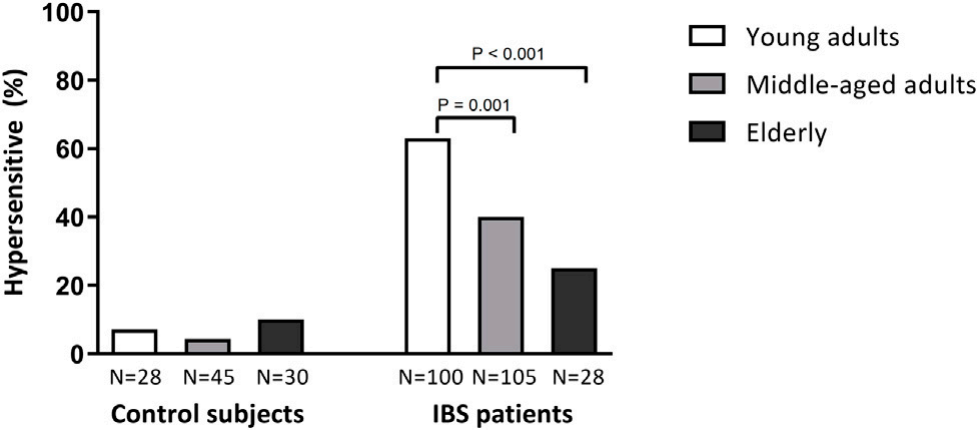
Occurrence of visceral hypersensitivity in healthy (control) subjects and IBS patients, based on rectal barostat data (semi-random staircase protocol) from the MIBS cohort. χ^2^ tests were used for comparing prevalence of visceral hypersensitivity per age group.

Visceral hypersensitivity was found to be less common among elderly IBS patients as compared to young adults and middle-aged patients (Figure 2). In the youngest age category ranging from 18–39 years, visceral hypersensitivity was found in 63.0% of IBS patients. In middle-aged adult (40–64 years) and elderly (65–75 years) IBS patients, visceral hypersensitivity was found in 40.0 and 25.0%, respectively. A χ^2^ test on all three age categories indicated a significant difference between at least two age groups (χ^2^ (1, N = 233) = 17.65, *p* < 0.001). Post-hoc testing revealed that visceral hypersensitivity was significantly more common in young-adults versus elderly (χ^2^ (1, N = 128) = 12.75, *p* < 0.001) and in young-adults versus middle-aged patients (χ^2^ (1, N = 205) = 10.85, *p* = 0.001). Overall low percentages of visceral hypersensitivity were found in all age groups of healthy controls (7.1, 4.4 and 10.0% respectively).

### TRPA1 and TRPV1 Immunoreactivity and mRNA Expression Profiles in Healthy Subjects

**FIGURE 3:**
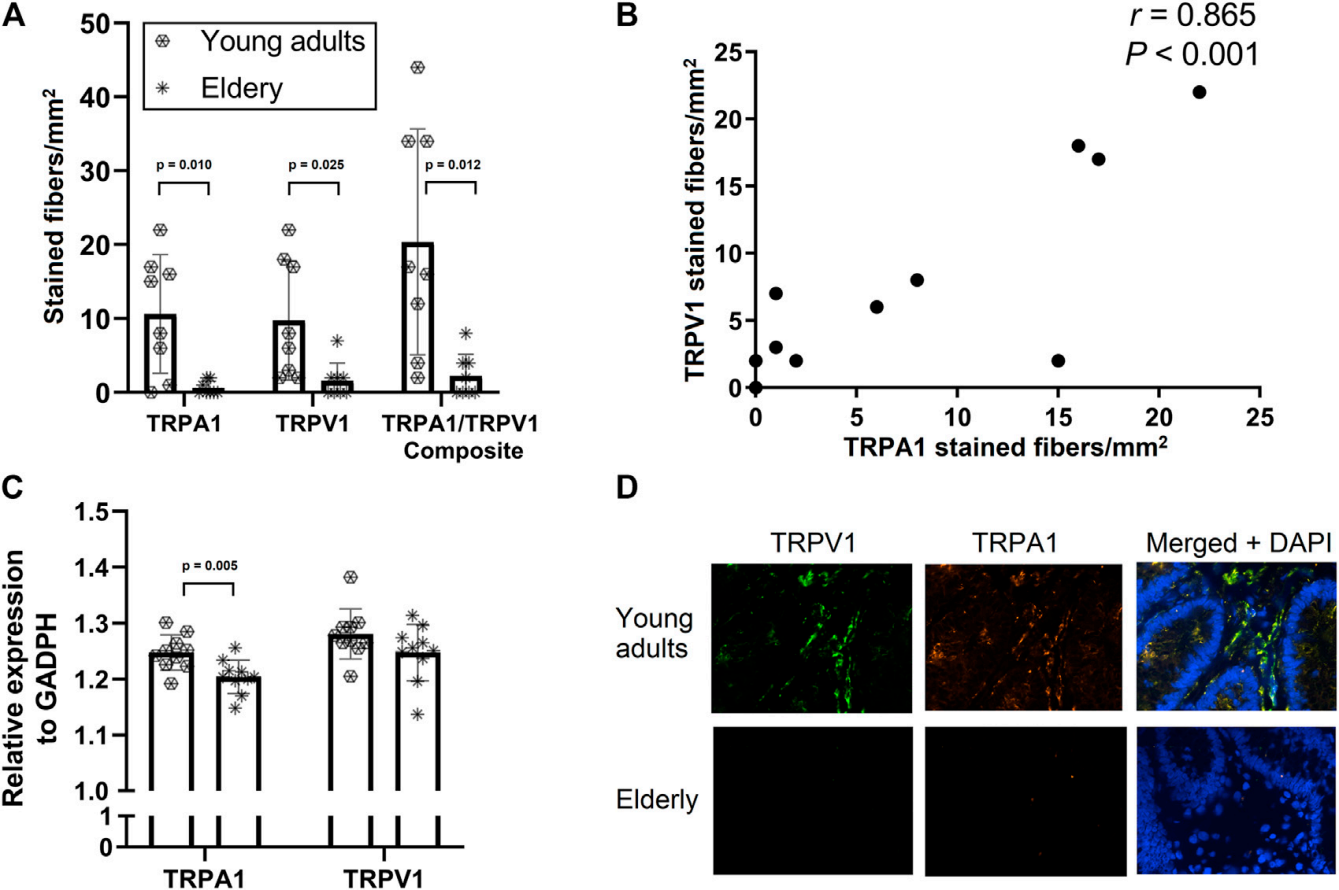
Panel **(A–D)**. Panel **(A)**: Immunoreactivity values (mean number of stained fibers per mm^2^ of lamina propria) of TRPA1, TRPV1 and combined (TRPA1/TRPV1 composite) of sigmoid biopsies from healthy young adults and healthy elderly. Panel **(B)**: Correlation of immunoreactivity of TRPA1 and TRPV1 (mean number of stained fibers per mm^2^ of lamina propria), demonstrating co-expression. Panel **(C)**: TRPA1 and TRPV1 expression (relative to GADPH as reference gene) in young adults and elderly. Panel **(D)**: immunofluorescence images demonstrating abundant staining of TRPV1 and TRPA1 in sigmoid biopsies from young adults, but not in the elderly. Abbreviations: DAPI = 4′,6-diamidino-2-phenylindole (cellular immunofluorescence stain).

TRPA1, TRPV1 and TRPA1/TRPV1 composite immunoreactivity was significantly lower in elderly subjects (Figure 3A). In addition, TRPA1 and TRPV1 immunoreactivity demonstrated a strong correlation (r = 0.865, *p* < 0.01, Figure 3B). Relative mRNA expression of TRPA1, but not TRPV1, was significantly lower in elderly subjects (Figure 3C). Representative immunofluorescence slides of TRPA1 and TRPV1 stainings in young adults and elderly are shown in Figure 3D. A moderate correlation was found between TRPA1 and TRPV1 relative mRNA expression levels, though this correlation failed to reached statistical significance (r = 0.442, *p* = 0.051, Supplementary Figure S1A). Immunoreactivity and relative mRNA expression levels correlated moderately for both TRPA1 (r = 0.530, *p* = 0.038, Supplementary Figure S1B) and TRPV1 (r = 0.566, *p* = 0.022, Supplementary Figure S1C. Abdominal pain scores correlated moderately with TRPV1 relative expression (r = 0.502, *p* = 0.024, Supplementary Figure S2B). No significant correlations were found between abdominal pain scores and TRPA1 relative expression, nor with TRPA1 and TRPV1 immunoreactivities (Supplementary Figure S2).

## Discussion

To the best of our knowledge this is the first study to explore associations between age-related changes in abdominal symptoms and TRPA1 and TRPV1 expression profiles in humans. First, our study corroborates the previously reported finding that abdominal pain decreases with age. Importantly, this decrease was not limited to healthy subjects, as it was also observed in a large group of IBS patients. We have previously reported that a significant proportion of IBS patients demonstrates lower gastrointestinal symptom scores over a follow-up period of 5 years as compared to their baseline values (Weerts et al., 2019). In the current study, we found that other gastrointestinal symptoms, such as diarrhoea and indigestion also decreased with ageing in IBS patients and healthy subjects, respectively. It should be noted that, based on these symptom reports alone, one cannot differentiate between an effect of ageing on visceral nociception and IBS natural disease progression. Hence, in the current study we have attempted to explore potential underlying mechanisms of decreasing abdominal pain scores with ageing in both IBS patients and healthy individuals.

In line with the decreased abdominal pain scores, we observed that visceral hypersensitivity measured by rectal barostat procedure, was significantly less common among elderly IBS patients than younger patients. This reduction was also apparent when comparing young adults and middle-aged IBS patients, pointing to a gradual decline in visceral sensitivity with age. Visceral hypersensitivity was an infrequent finding in healthy volunteers in all age groups, hence no decline with age was observed in this subgroup of healthy controls.

In a subgroup of healthy subjects, we used immunofluorescence to measure TRPA1 and TRPV1 protein expression in sigmoid biopsies of both healthy young adults and elderly subjects. These TRP channels have been implicated to play important roles in the transduction of nociceptive information from the gut and several studies have shown upregulation of TRPV1 in the colonic mucosa of IBS patients (Beckers et al., 2017). TRP channels have therefore been implicated as potential therapeutic targets for the treatment of chronic abdominal pain. Here we found, for the first time in human colonic tissue, that TRPA1 and TRPV1 immunoreactivity was significantly lower in biopsies from healthy elderly subjects compared to their younger counterparts. Given the role of these molecular transducers in visceral nociception, we subsequently explored associations between TRPA1 and TRPV1 immunoreactivity and abdominal pain scores. Only TRPV1 mRNA expression correlated moderately with abdominal pain. No significant correlations were found between abdominal pain scores and TRPA1 mRNA expression, nor with immunoreactivity of TRPA1 or TRPV1. It should be pointed out, however, that abdominal pain scores were rather low in this specific subgroup of healthy subjects, with a maximum pain score of only 3 (on a 7-point Likert scale.

Another important observation is the strong correlation between TRPA1 and TRPV1 immunoreactivity, which is in line with previous findings indicating that TRPA1 is almost exclusively expressed on TRPV1-positive neurons (Beckers et al., 2017).

Using quantitative RT-PCR, we found that TRPA1, but not TRPV1, mRNA expression levels were lower in elderly subjects. The discrepancy between the protein and mRNA expression levels can in part be explained by the fact that PCR encompasses TRP mRNA from all cellular origins, including non-neuronal (*e.g.* epithelial), whereas for immunofluorescence staining was predominantly quantified for (morphologically defined) neural fibres. Moreover, not all mRNA is converted into protein and this may also be affected by ageing (*i.e.* mRNA decay). The latter was also found in a mouse study, where TRPV1 protein expression decreased by ageing in dorsal root ganglion (DRG) neurons, but TRPV1 mRNA expression levels were not altered (Wang et al., 2006). In line with these findings, we observed only modest (but statistically significant) correlations between immunoreactivity and expression levels from the same TRP channel (Supplementary Figure S1B,C). Another question is to which extent neural elements are affected by potential mRNA decay. Findings in literature are somewhat incongruent in this respect. Cibert-Goton *et al.* found decreased afferent responses to capsaicin, but only in multi-unit and not in single-unit recordings (Cibert-Goton et al., 2020). In addition, McGuire *et al.* found no effect of age on single unit responses to von Frey hair probing, bradykinin or ATP (McGuire et al., 2018). On the other hand, Keating *et al.* showed that the decreased response to capsaicin was observed in single-unit recordings, which would rather suggest age-related loss in TRPV1 channel function.

It has been postulated that neurodegeneration, in particular of the enteric nervous system, plays a key role in the deterioration of gut functioning with increasing age (Saffrey, 2013; Soenen et al., 2016). Indeed, the ENS regulates most gastrointestinal functions, ranging from absorption and secretion to motility, afferent signaling, and also immune and inflammatory responses. Thus, decreased functioning of the ENS would reflect on all these functions. A limitation of the current study is that we did not obtain specific neural stainings in the biopsy materials. It is therefore unclear whether the decreased TRPV1 and TRPA1 immunoreactivity was due to decreased expression on afferent fibers, or due to neurodegeneration (*i.e.* lower density of neural fibers). The distinction is particularly difficult to make for gastrointestinal biopsy specimens, as TRPV1 and TRPA1 themselves are not solely involved in nociception, but have been implicated in visceral motility and permeability as well (Beckers et al., 2017). In order to obtain a complete view of the enteric nervous system one would require intestinal resection material instead of mucosal biopsy specimens.

When exploring associations between TRPA1 and TRPV1 expression levels and abdominal pain scores, a moderate correlation was found for TRPV1 expression only. One should realise that pain is a highly complex mechanism, as not only biological factors (*e.g.* neural density and afferent functioning), but also psychological factors (*e.g*. cognitions and stress) play a substantial role in the ultimately perceived sensation after an applied stimulus. As a result, there is a non-linear relationship between peripheral nociceptive input and pain sensation. Cognitions about the meaning of pain (and other abdominal symptoms) as well as coping strategies may be particularly important in the context of decreased abdominal symptoms with ageing. Unfortunately, these factors are rather subjective and therefore, difficult to measure, and as such, have not been included in the present study. Although one can only speculate about the importance of altered coping strategies with ageing, our study provides evidence that decreased sensory function of the gut involves biological changes in visceral afferents, as indicated by the decreased TRPA1 and TRPV1 expressions. Changes in abdominal symptoms over long periods of time may however also be related to habituation (Ginzburg et al., 2015), especially since our elderly IBS patients reported a significant longer duration of IBS symptoms compared to the younger patient groups. Nevertheless, such an effect is unlikely to play a role when using the rectal barostat procedure, which was novel to all subjects. We therefore think that further research is warranted for investigating the association between reduced abdominal symptoms and visceral hypersensitivity with age, and changes in neural TRP channel expression profiles. The latter should also account for neural density, as current evidence cannot rule out that the decreased nociceptive sensitivity could be driven by neurodegeneration rather than specific alterations in afferent functions. In this sense, age-related changes in nociceptive signaling can potentially have beneficial effects in patients experiencing chronic pain as a result of nociceptive overdrive or other forms of visceral hypersensitivity.

Another limitation of our paper is that we did not obtain biopsies from IBS patients. Had we done so, we could have assessed whether there are any age-related changes in expression profiles that are specific to IBS. Therefore, we consider our study exploratory in nature and further studies will be warranted to examine exact underlying mechanisms in different (patient) populations. Results presented in the current study need to be interpreted in light of the exploratory nature. This is particularly important with our biopsy results, where sample sizes (and as such statistical power) were limited. Finally, one should keep in mind that data from two separate studies was used, which poses methodological limitations (e.g. variability in subject populations).

Understanding the processes of the age-related decrease of the intensity of abdominal sensations can have important therapeutic repercussions. First, the decreased perception of adverse stimuli is beneficial for patients with chronic abdominal pain, such as in IBS. Given this observation, patients can be reassured that their symptoms are likely to diminish over time. Moreover, insights regarding this natural analgesic effect of ageing could prove valuable for the development of novel (visceral) pain management strategies.

## Conclusion

An age-related decrease in the reporting of abdominal pain is found in both IBS patients and healthy individuals. Furthermore, the prevalence of visceral hypersensitivity in IBS patients decreases with age. This may be attributed to biological changes, such as decreased TRPA1 and/or TRPV1 receptor expression in the intestinal epithelium. Further studies are needed to specify the underlying mechanisms and the association with intestinal health.

## Data Availability

The original contributions presented in the study are included in the article/Supplementary Materials, further inquiries can be directed to the corresponding author.
