# Supplementary material for: Age-Related Decrease in Abdominal Pain and Associated Structural- and Functional Mechanisms: An Exploratory Study in Healthy Individuals and Irritable Bowel Syndrome Patients
Source: Front Pharmacol. 2021 Dec 16;12:806002. doi: 10.3389/fphar.2021.806002 (PMC8716827; doi:10.3389/fphar.2021.806002)
Supplement: Supplementary file 1 [file DataSheet1.PDF]

Supplementary Material

Supplementary Table 1. Mean GSRS scores of IBS patients (MIBS cohort) and healthy subjects (MIBS cohort and biopsy study).

	**IBS patients (MIBS)**			**Healthy subjects (MIBS)**			**Healthy subjects (biopsy study)**	
	**Young adults**	**Middle-aged**	**Elderly**	**Young adults**	**Middle-aged**	**Elderly**	**Young adults**	**Elderly**
N	191	209	63	90	84	43	52	48
Abdominal pain (mean ± SD)	3.64 ± 1.22***	3.19 ± 1.18	2.91 ± 1.18	1.93 ± 0.81***	1.49 ± 0.62	1.37 ± 0.42	2.03 ± 0.70***	1.43 ± 0.61
Reflux (mean ± SD)	2.16 ± 1.36	2.23 ± 1.44	1.98 ± 1.34	1.24 ± 0.51	1.20 ± 0.57	1.22 ± 0.48	1.29 ± 0.60	1.19 ± 0.60
Diarrhoea (mean ± SD)	3.83 ± 1.54**	3.16 ± 1.51	3.07 ± 1.51	1.51 ± 0.74	1.32 ± 0.50	1.36 ± 0.59	1.54 ± 0.79	1.46 ± 0.66
Indigestion (mean ± SD)	4.21 ± 1.26	3.99 ± 1.32	3.75 ± 1.48	2.22 ± 0.85*	1.94 ± 0.91	1.75 ± 0.85	2.26 ± 0.79***	1.67 ± 0.78
Constipation (mean ± SD)	3.49 ± 1.42	3.12 ± 1.39	3.03 ± 1.22	1.66 ± 0.91	1.58 ± 0.79	1.57 ± 0.82	1.62 ± 0.72	1.49 ± 0.73

* $P < 0.05$ ** $P < 0.01$ *** $P < 0.001$ (vs elderly for MIBS cohort)
SD = standard deviation
Independent t-tests were used for comparison of mean GSRS scores per age group
MIBS age groups: 18-39 years (young adults), 40-64 years (middle-aged adults), 65-75 years (elderly)
Biopsy study age groups: 40-64 years (middle-aged adults), 65-75 years (elderly)

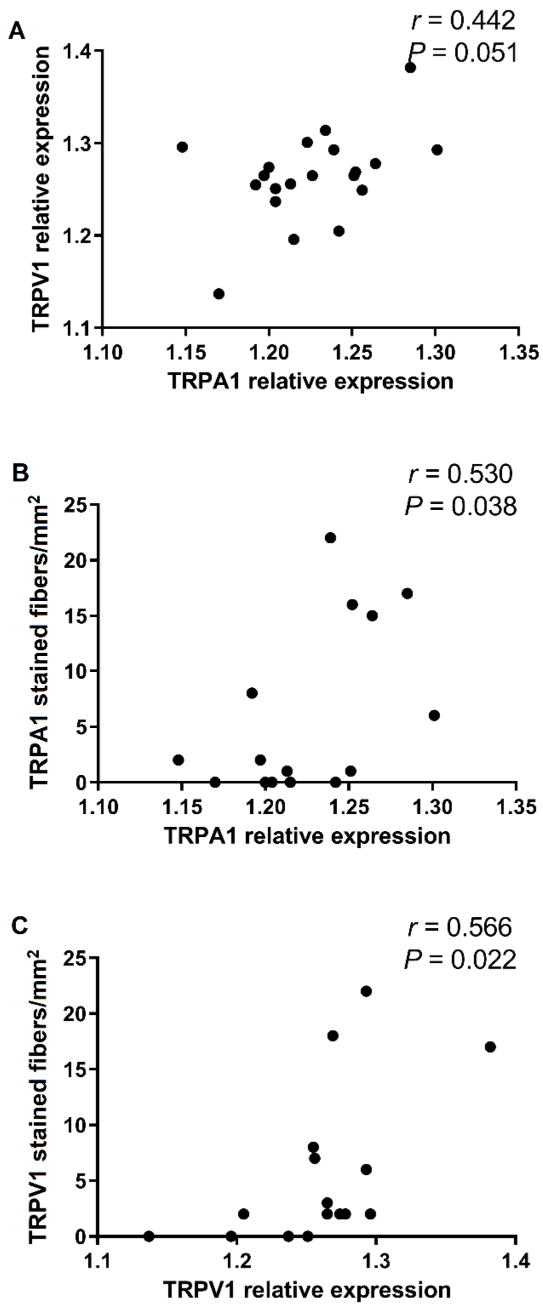

Supplementary Figure 1 (panel A-C). Panel A: Correlation between TRPA1 relative expression and TRPV1 relative expression. Panel B: Correlation between TRPA1 immunoreactivity (mean number of stained fibers per mm^2 of lamina propria) and TRPV1 relative expression. Panel C: Correlation between TRPV1 immunoreactivity and TRPV1 relative expression.

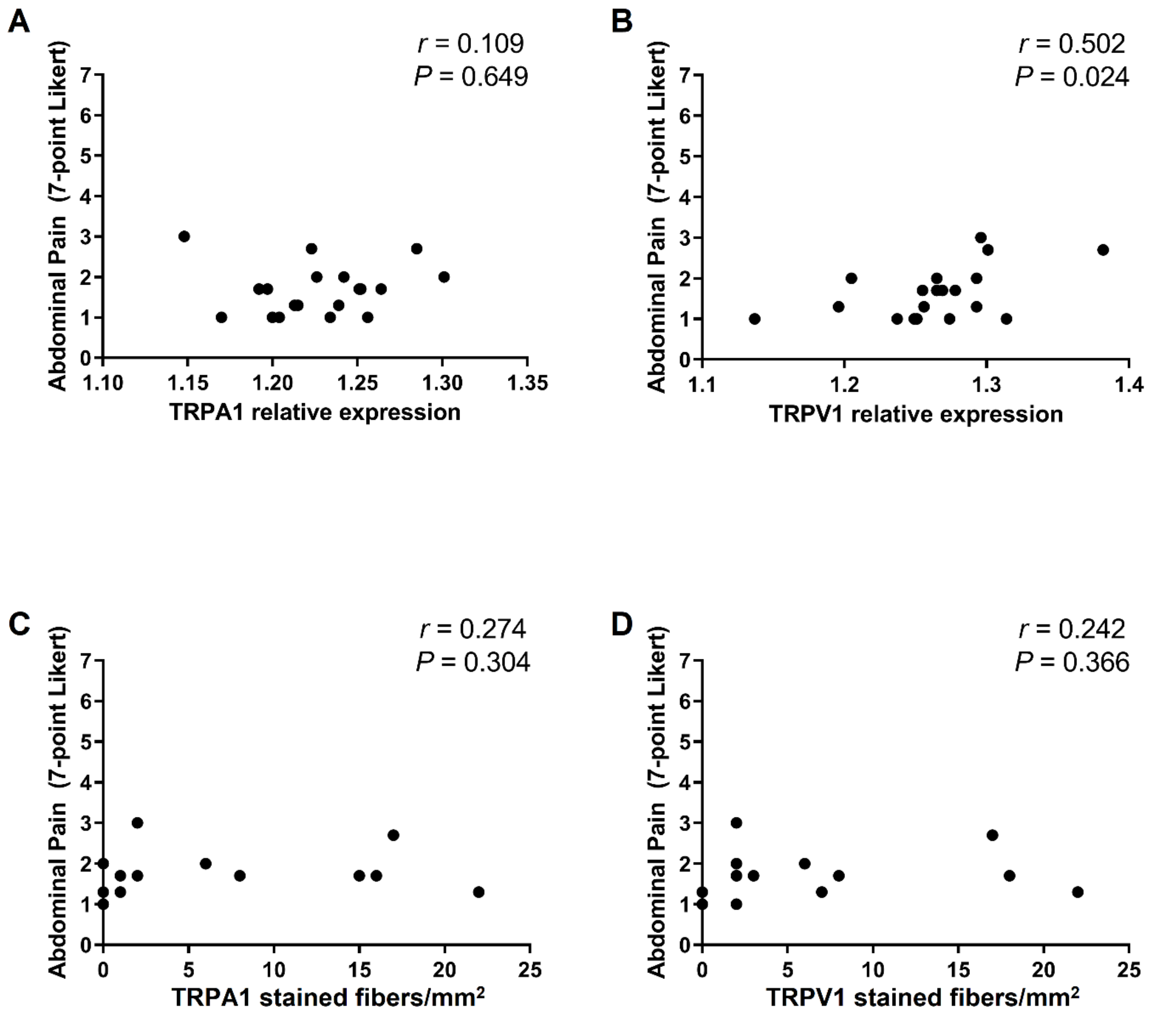

Supplementary Figure 2 (panel A-D). Correlation between abdominal pain and A) TRPA1 relative expression, B) TRPV1 relative expression, C) TRPA1 immunoreactivity (mean number of stained fibers per mm^2 of lamina propria) and D) TRPV1 immunoreactivity.
